# Genetic Counseling for Hereditary Gastric and Pancreatic Cancer in High-Risk Gastrointestinal Cancer Clinics: An Effective Strategy

**DOI:** 10.3390/cancers12092386

**Published:** 2020-08-23

**Authors:** Joan Llach, Lorena Moreno, Ariadna Sánchez, Cristina Herrera-Pariente, Teresa Ocaña, Miriam Cuatrecasas, Liseth Rivero-Sánchez, Rebeca Moreira, Mireia Díaz, Gerhard Jung, Maria Pellisé, Antoni Castells, Francesc Balaguer, Sabela Carballal, Leticia Moreira

**Affiliations:** 1 Gastroenterology Department, Hospital Clínic de Barcelona, Centro de Investigación Biomédica en Red de Enfermedades Hepáticas y Digestivas (CIBERehd), Institut d’Investigacions Biomediques August Pi i Sunyer, 08036 Barcelona, Spain; JLLACHR@clinic.cat (J.L.); LOMORENO@clinic.cat (L.M.); asanchezg@clinic.cat (A.S.); cristina.herrera@ciberehd.org (C.H.-P.); MOCANA@clinic.cat (T.O.); LRIVERO@clinic.cat (L.R.-S.); RMOREIRA@clinic.cat (R.M.); MDIAZC@clinic.cat (M.D.); JUNG@clinic.cat (G.J.); mpellise@clinic.cat (M.P.); CASTELLS@clinic.cat (A.C.); fprunes@clinic.cat (F.B.); carballal@clinic.cat (S.C.); 2 Pathology Department, Centre for Biomedical Diagnosis, Hospital Clínic, 08036 Barcelona, Spain; mcuatrec@clinic.cat

**Keywords:** gastric cancer, pancreatic cancer, hereditary cancer, familial cancer, genetic counselling

## Abstract

The identification of high-risk groups of gastric (GC) and pancreatic adenocarcinoma (PC) due to a hereditary basis could imply a benefit in the affected families by establishing personalized preventive strategies. We aimed at assessing the diagnostic yield of GC/PC hereditary syndromes in individuals evaluated based on specific clinical criteria. In total, 77 unrelated individuals (45 from GC group/32 from PC group) were recruited: 51 (66.2%) cancer diagnosis ≤60 years, 3 (4%) with personal history of GC/PC and other cancer and 23 (29.8%) due to family history. Immunohistochemical analysis of DNA mismatch repair proteins was performed in 38 (49.3%) available tumors, being pathological in one (2%) GC. A genetic analysis was performed if clinical criteria of hereditary syndrome were fulfilled, identifying a mutation in 10/22 (45.5%) families [7/16 (43.7%) with GC and 3/6 (50%) with PC] and 19 (24.7%) fulfilled criteria of familial cancer. Diagnosis of cancer <40 years and personal history of other cancers were independent risk factors of a hereditary syndrome [OR:11.3 (95%IC 1.9–67); *p* = 0.007 and OR:17.4 (95% IC 2.5–119.9); *p* = 0.004; respectively]. The selection of patients based on clinical criteria leads to high diagnostic yield, detecting a causative germline mutation in almost half of the cases; therefore, both meticulous genetic counseling and use of multi-gen panels is crucial.

## 1. Introduction

Genetic counseling in the setting of gastrointestinal malignancies has been focused on the identification of hereditary colorectal cancer, and particularly in Lynch Syndrome, the most frequent inherited form of colon cancer [1,2,3].

However, familial aggregation and hereditary component in cases of extra-colonic gastrointestinal tumors, such as gastric adenocarcinoma (GC) and pancreatic ductal adenocarcinoma (PC), have been less studied. These tumors represent a relevant health problem in developed countries due to their poor prognosis, mainly because most of them are diagnosed in advanced stages. Thus, according to GLOBOCAN 2018 data, GC represents the fifth most common cancer and third leading cause of cancer deaths worldwide. PC is the fifth leading cause of cancer death in Europe, and the seventh globally; in fact, it is expected to become the second leading cause of cancer death worldwide by 2030 [4,5].

In about 10% of all GC or PC, there is a hereditary component, either because there is a germline mutation (i.e., hereditary cancer), or, in the absence of a germline variant, familial aggregation of these tumors is observed (i.e., familial cancer) [6,7,8,9]. Familial GC (FGC) is defined as ≥3 first- or second-degree relatives (FDR and SDR, respectively) with GC or ≥2 FDR/SDR with GC (at least one diagnosed <50 years of age). Familial PC (FPC) is defined as ≥2 FDR with PC or ≥3 relatives with PC, regardless of the degree and age of the relative [9]. In clinical practice, in depth personal and family oncological history in cases of GC or PC is unusual, which implies that many tumors that could have a hereditary component or family aggregation are misclassified as sporadic, and, therefore, specific preventive measures are not applied to family members [10].

Hereditary diffuse gastric cancer syndrome (HDGC) [11] is the most common inherited entity related to GC, involving a germline mutation in *CDH1* and much less frequently in *CTNNA1* [12], with a cumulative risk of GC of up to 80%. Besides this syndrome, there are other genes associated with an increased risk of GC: *STK11* (Peutz–Jeghers syndrome), *SMAD4*/*BMPR1A* (juvenile polyposis), *MLH1*/*MSH2*/*MSH6*/*PMS2* (Lynch syndrome), *APC*/*MUTYH* (familial adenomatous polyposis), *BRCA2*/*BRCA1*/*PALB2/ATM* (hereditary breast-ovarian cancer syndrome, HBOC) and *TP53* (Li-Fraumeni syndrome) [13,14,15].

Regarding PC, there is no main causative gene for PC predisposition, but several known hereditary syndromes are related with an increased risk of this tumor [16]. The most frequent germline genetic alterations associated with PC are present in *BRCA2*, *PALB2*, *ATM* (ataxia telangiectasia) and *CDKN2A*/*p16* (familial atypical multiple mole melanoma, FAMMM) and, less frequently, *BRCA1*, *APC*, *MLH1*, *MSH2*, *MSH6*, *PMS2*, *PRSS1* (hereditary pancreatitis) and *STK11* [17].

Recent studies suggest that screening programs in high-risk groups for these malignancies (normally defined as life-time cumulative risk over 5%) may increase survival in those specific groups [18]. The identification of hereditary and familial forms of GC or PC could imply a benefit in the affected families, as they can be included in prevention and surveillance programs adapted to their intrinsic risk [6,19,20,21,22,23].

With the final goal of improving survival associated with GC or PC through its prevention and early diagnosis, we aimed at evaluating the diagnostic yield in identifying high-risk forms of GC or PC (Hereditary or familial GC or PC) in a HRC of gastrointestinal cancer.

## 2. Material and Methods

This study was conducted at the HRC of gastrointestinal cancer of the Hospital Clínic of Barcelona. Patients were referred from primary care doctors, as well as oncologists, gastroenterologists and surgeons of the hospital.

**Figure 1:**
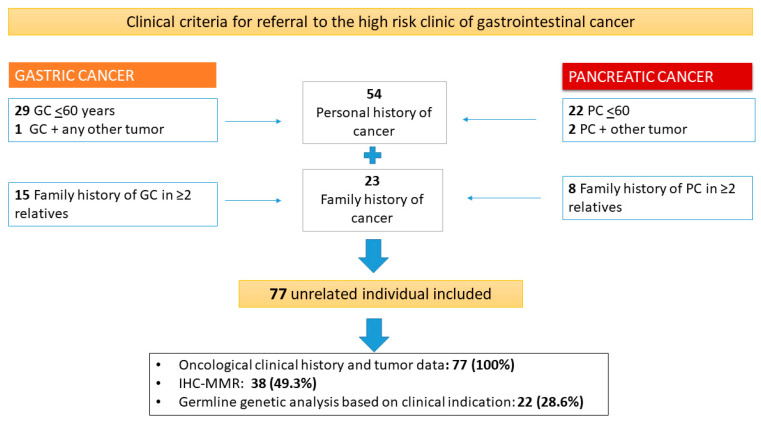
Counseling process at the high-risk clinic of gastrointestinal cancer for diagnosis of GC or PC familial or hereditary syndromes. In the high-risk clinic for gastrointestinal cancer, all patients with GC or PC were evaluated at a young age (≤60) or at any age if they also had another tumor; healthy individuals who had a family history of one of these malignancies were also evaluated. After counseling and molecular or genetic analyzes if it was indicated, all families were classified as a form of hereditary, familial or sporadic cancer. GC, gastric cancer; PC, pancreatic cancer; IHC-MMR, Immunohistochemical analysis of DNA repair proteins.

Clinical criteria for referral of suspected hereditary and familial GC and PC were defined as follows: (a) an individual with a history of GC or PC before 60 years of age; (b) an individual with personal history of GC or PC (at any age) and any other malignancy; and (c) healthy individuals with a family history of cancer defined as >2 relatives with GC or >2 relatives with PC, regardless of the degree and age of the relative (Figure 1).

In all cases, personal and familial oncological history was collected, and the characteristics of the tumors were analyzed. Immunohistochemical analysis of DNA mismatch repair proteins (IHC-MMR): MLH1, MSH2, MSH6 and PMS2 was performed in the available tumors, as previously described [24]. In patients with clinical criteria and available DNA, a germline genetic analysis was performed. Most of the patients received a multigene testing, by simultaneous sequencing through a commercial multigene panel (Trusight Cancer v1, Illumina Inc., San Diego, CA, USA), involving the most frequent genes related with GC and PC germline predisposition. In a few cases, a single gene analysis was carried based on clinical suspicion and specific phenotype (i.e., guided by a pathological IHC-MMR observed in the analyzed tumor).

Finally, “hereditary” GC or PC was classified when a germline pathogenic variant was detected and “familial” GC or PC when the established and current clinical criteria were met and no germline mutation was present.

Statistical analysis was performed using SPSS 22.0 (IBM Corp., Armonk, New York, NY, USA). Quantitative variables are expressed as medians and interquartile ranges (IQRs) and categorical variables are expressed as total number and frequencies (%). Quantitative variables were analyzed using Student’s *t* test and qualitative variables were analyzed using the chi-squared test.

Univariate binary logistic regression was performed for selection of variables associated with the diagnosis of a hereditary/familial cancer. For multivariable logistic regression analyses, only candidate variables with statistically significant *p* values (defined as *p* < 0.05) on univariate analysis were used in the final multivariate model. Odds ratios (ORs) with 95% confidence intervals (CIs) were included to quantify the magnitude of the association.

The present study was approved by the Institutional Ethics Committee (For GC cohort: register number HCB/2019/0408 and date of approval 02/05/2019; for PC cohort: register number HCB/2020/0298 and date of approval 26/03/2020). Written informed consent was obtained in all cases.

## 3. Results

### 3.1. Characteristics of the Studied Population

Between May 2014 and October 2019, 77 unrelated individuals that met the established criteria for referral were evaluated: 45 (58.4%) due to personal and/or family history of GC and 32 (41.6%) due to personal and/or family history of PC.

In relation to the clinical criteria, the majority of patients (51 [66.2%]) were referred for GC or PC diagnosed before the age of 60; 3 (4%) patients for personal history of GC or PC and another neoplasm (corresponding in these 3 specific cases to breast, colon and endometrium); and 23 (29.8%) were healthy individuals referred due to GC or PC family history (Figure 1).

Considering the youngest age of diagnosis of GC or PC in each family, the median age was 49 years old (IQR: 41.5–58), slightly younger in cases of GC than PC: 47 vs. 51.5 (*p* = 0.06). The distribution by gender was similar, with 46 (59.7%) evaluated women [29 (64.4%) in GC and 17 (53.1%) in PC].

Nine of the 77 (11%) individuals evaluated had previous personal history of other type of cancer (five breast cancers, two endometrial cancers, one colorectal cancer and one lymphoma). Within the entire cohort, 53 (68.8%) reported family history of other malignancies, with a median of two (IQR: 1–3) malignancies in first- and second-degree relatives. Of the 121 registered malignancies, breast (18), colon (13) and prostate (6) were the most frequently affected organs. Detailed clinical characteristics of the evaluated individuals are shown in Table 1.

### 3.2. Mismatch Repair Deficiency and Germline Genetic Analysis

Immunohistochemistry for MLH1, MSH2, MSH6 and PMS2 (IHC-MMR) was performed on paraffin-embedded samples of 29 gastric and 9 pancreatic tumor tissues, being altered in one (2.6%) of the 38 analyzed tumors. The sample with MMR deficiency involved a gastric tumor with intestinal-type histology in a 75-year-old woman. The patient had previous history of endometrial cancer at 53 years (Table 2, Patient 1). The gastric tumor showed loss of expression of MSH2 and MSH6 proteins and the germline genetic analysis detected a pathogenic variant in *MSH2* (c.602dupT p. Leu201Phe*31), leading to the diagnosis of Lynch syndrome. The patient had a daughter with endometrial cancer at 50 year and there was no family history of gastric or any other tumor.

Germline genetic study was conducted in 22 (28.6%) patients with available DNA [16/45 (35.6%) patients with GC and in 6/32 (18.8%) patients with PC]. A guided study focused on the suspected gene was performed in 9 (41%) and a multi-gene panel in 13 (59%) cases. Regarding PC genetic counseling, only one guided genetic study was performed (based on clinical criteria of hereditary breast and ovarian cancer syndrome, HBOC; Table 2, Patient 8), and a multi-gene panel was used in the five other cases. In families evaluated due to GC history, a directed study was carried out in half of the cases and a multi-gene panel in the other half.

A causative germline variant was detected in 10 cases (representing 13% of the entire cohort, and 45.5% of the patients with germline test done).

**Figure 2:**
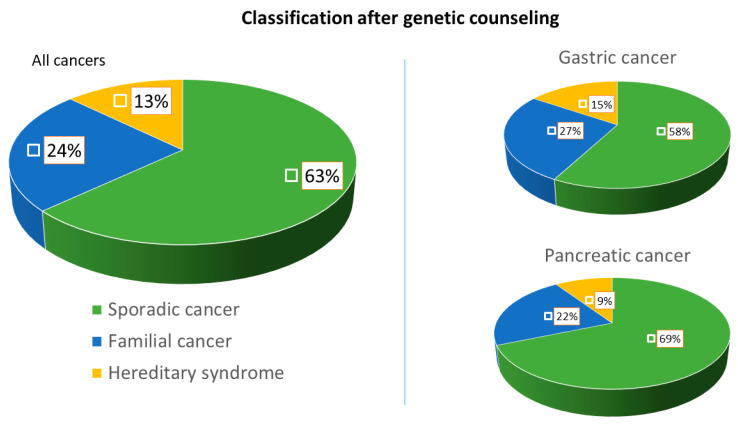
Final diagnostic classification of the families evaluated at the HRC of gastrointestinal cancer. This figure represents the percentage distribution of the families evaluated in three final diagnostic categories: (1) “hereditary syndrome” (cancer attributable to a germline genetic mutation); (2) “familial cancer” (due to compliance with current clinical criteria); and (3) “sporadic cancer” (attributable to environmental factors that exclude the hereditary component).

Within the GC group, seven cases (representing 15.5% of the GC cohort and 43.7% of the GC patients with germline test done) were diagnosed with a hereditary syndrome: one Lynch syndrome (*MSH2*), two ATM hereditary cancer syndromes (*ATM*), two HDGC (*CDH1*), one HBOC (*BRCA2*) and one Li–Fraumeni syndrome (*TP53*). Regarding the PC group, three patients (representing 9.3% of the PC cohort and 50% of the PC patients with germline test done) were diagnosed with an inherited syndrome: two HBOC (1 *BRCA2* and 1 *PALB2*) and one *ATM* hereditary cancer syndrome (*ATM*). The germline pathogenic variants detected and the characteristics of the families affected are specified in Table 2. In addition, 19 (24.6%) families met the criteria for a familial cancer: 12 (26.6%) FGC and 7 (21.8%) FPC (Figure 2).

### 3.3. Performance of Clinical Criteria for the Detection of High-Risk Forms

Analysis of association between baseline characteristics and clinical criteria for detecting high-risk forms of GC and PC is represented in Table 3.

The criterion “age ≤60” was the most frequent reason for referral (51 patients; 66.2%). In this group, the median age at cancer diagnosis was 47 years (IQR 40–50). A genetic study was performed in 12 (23.5%) cases. Following genetic counseling, 42 (82.7%) of the cases were classified as “sporadic cancer”, 6 (11%) had a hereditary syndrome and 3 (5.8%) a familial form of cancer. Age ≤60 was not associated with a higher probability of detecting high-risk forms of PC or GC (Table 3).

The “multiplicity” criterion (having GC or PC and any other neoplasm) only motivated the referral of three (3.9%) patients. In one case, previously mentioned (Table 2, Patient 1) it was with endometrial and gastric cancer diagnosed of Lynch Syndrome. The other two cases corresponded to two women diagnosed with PC at 68 and 67 years of age, respectively. The first one had breast cancer at age 53 and the second one had breast cancer at age 60 and colon cancer at 63. In both cases, a germline genetic study was performed using a multi-gene panel, including the analysis of DNA-MMR and *BRCA*-like genes. No pathogenic mutation was found and both cases were classified as sporadic.

Lastly, 23 (29.9%) cases were referred based on family history: 19 (82.6%) were finally classified within the familial cancer category and 4 (17.4%) as sporadic tumors. No association between meeting this criterion and a higher probability of detecting a hereditary syndrome was observed, but it proved to be an independent risk factor for detecting a high-risk form [OR: 21 (95% CI 5.8–79); *p* = 0.000].

Other baseline factors were analyzed, such as age, sex and personal and family history of any other malignancies. In the univariate analysis, a significantly younger age of cancer diagnosis was observed in families with hereditary syndrome compared to sporadic cases (median age 39.5 vs. 50 years; *p* = 0.04). Otherwise, in sporadic cancer cases, only 15% of tumors occurred at <40 years, versus a 50% of hereditary cases (*p* = 0.009). Age <40 years proved to be an independent risk factor for the diagnosis of a hereditary syndrome in the multivariate analysis [OR: 11.3 (95% confidence interval: 1.9–67); *p* = 0.007].

On the other hand, although the “multiplicity” criterion was only the reason for referral in three cases in the entire series, nine individuals had a personal history of GC/PC and another malignancy and four of them (44.4%) had a hereditary syndrome. Thus, the personal history GC/PC and any other neoplasm in these patients also proved to be an independent risk factor for the detection of an inherited syndrome [OR: 17.4 (95% confidence interval: 2.5–119.9); *p* = 0.004].

## 4. Discussion

This study represents the first series of gastric or pancreatic cancer individuals systematically evaluated with the aim of assessing the efficacy of suspicious clinical criteria in identifying high-risk forms of these tumors in clinical practice. The results demonstrate the effectiveness of this strategy, identifying a hereditary syndrome or a familial form of cancer in 37% of the families evaluated.

GC and PC are two of the most lethal tumors. In both cases, prevention measures focused on the general population have shown low cost-effectiveness but they could have a role in high-risk forms of cancer [6,7,8,9]. Those individuals in whom a genetic mutation is identified (hereditary cancer) and those in which GC/PC family aggregation is observed despite the identification of an underlying genetic mutation are currently recognized as “high-risk forms”. According to previous literature, this situation is responsible for 10% of all GC and PC [25,26,27,28,29].

Our study attempted to assess effectiveness in identifying these high-risk forms through actively searching for suspected cases based on pre-selected clinical criteria. These criteria took into account the age of presentation of the tumor, the association with other tumors and the aggregation of several cases of the same type of malignancy in a single family.

After including 77 unrelated individuals during five years, we observed that more than 30% of the cases evaluated belonged to one of these high-risk forms. Given that both GC and PC are part of the spectrum of tumors associated with Lynch syndrome, and the diagnostic accuracy of IHC-MMR in paraffin-embedded samples is well established to rule it out [30], this molecular technique was performed on all available tumors. An alteration in protein expression was only detected in one of the 38 gastric tumors analyzed and in none of the 19 PC analyzed, suggesting that the IHC-MMR has a minor role in identifying inherited forms of GC and PC.

On the other hand, a germline genetic study was carried out in all patients that fulfilled criteria of a hereditary syndrome based on personal and/or family history and according to current clinical guidelines. With this approach, a responsible genetic mutation was detected in 45.5% of the cases analyzed (43.7% of the families with GC and 50% of those analyzed with PC). A great variability was observed in the genes responsible for the observed phenotypes, suggesting that these tumors can appear in the setting of different hereditary forms of cancer, normally related to the development of other types of malignancies.

We analyzed the diagnostic yield in identifying high-risk forms of GC or PC based on specific clinical criteria, as well as diagnostic risk factors of hereditary cancer. The family aggregation criterion proved to be useful for the detection of family forms of cancer and the age below 40 years and the personal history of another neoplasm (associated with any of the referral criteria) were shown as independent diagnostic risk factors of hereditary cancer.

These results highlight the importance of carrying out a complete anamnesis of the personal and family history of tumors, as well as an adequate knowledge of hereditary forms of cancer for optimal genetic counseling. Furthermore, the variability of the observed syndromes reinforces the need to use multigene panels.

The main strength of the present study is that, to our knowledge, it is the first study in our setting that evaluates the effectiveness of genetic counseling in detecting hereditary or familial forms of gastric and pancreatic cancer. In addition, it has been developed in a high-risk clinic formed by professionals having a wide experience in the management of inherited syndromes associated with gastrointestinal cancer.

However, this study has some limitations: first, only 77 families were evaluated; thus, the size of the sample is not large enough to give sufficient power to the observed results. Despite this limitation, it is the largest published cohort, so the results obtained could be a good estimate of reality and stands the basis for future studies involving a larger number of families. Another limitation is that the data obtained were collected retrospectively, which implies potential inclusion biases. This limitation is hardly avoidable since family history is retrospective by definition and supposes an inherent condition of the study design. To mitigate its effect on the results, all the data obtained were carefully evaluated by experienced physicians. Finally, the patients and families were selected based on arbitrary criteria, probably too broad regarding the age cut-off and family history, and we did not perform germline genetic test on the entire cohort to extrapolate the results. However, these criteria were selected and agreed by several members of a multidisciplinary team to achieve a high sensitivity to detect the maximum number of candidate families to be included in prevention programs.

## 5. Conclusions

Our study shows that almost 40% of the families evaluated in the HRC of gastrointestinal cancer based on personal or family history of GC and PC have a high risk of cancer. On the other hand, although mismatch repair deficiency analysis seems to be unhelpful in this scenario, the genetic study based on clinical criteria detects a responsible genetic mutation in almost half of the patients. The results obtained in this series suggest that the age of cancer below 40 years or the personal history of other tumors associated with fulfillment of any of the established referral criterion are associated with a higher diagnostic probability of an inherited syndrome.

The spectrum of mutations detected in our series indicates that these two tumors occur within a wide range of hereditary syndromes, which reinforces the role of genetic counseling and suggests the need to apply multigene panels. We consider these results relevant given that recent studies show that specific screening strategies in high-risk groups for these tumors may imply an increase in survival. However, to extrapolate these conclusions, it is necessary to carry out prospective studies with larger cohorts to validate the proposal strategy.

## Figures and Tables

**Table 1:** Characteristics of the individuals evaluated at the high-risk gastrointestinal cancer clinic.

Characteristic	Gastric Cancer (45)	Pancreatic Cancer (32)	All (77)
**Age at cancer diagnosis**^1^; median (IQR)	47 (40–55)	51.5 (45.5–59)	49 (41.5–58)
**Gender** (women); number (%)	29 (64.4)	17 (53.1)	46 (59.7)
**Race**			
Caucasian, number (%)	43 (95.6)	33 (100)	75 (97.4)
Asian, number (%)	2 (4.4)	0	2 (2-6)
**Referral criteria, number (%)**			
-Age ≤60 years old	29 (64.4)	22 (68.5)	51 (66.2)
-Multiplicity (PH GC/PC and any other tumor)	1 (2.3)	2 (6.5)	3 (3.9)
-Family history	15 (33.3)	8 (25)	23 (29.9)
**PH of any other neoplasm**, number (%)	6 (13)	3 (8.8)	9 (11.7)
Age; median (IQR)	49 (41.5–58)	53 (35–53)	53 (39.5–58)
**Neoplasm type**, number (%)			
Breast	3 (50)	2 (66%)	5 (55.5)
Endometrium	2 (33.4)	-	2 (22.2)
Colon	1 (16.6)	-	1 (11.1)
Lymphoma	-	1 (34%)	1 (11.1)
**Family history of the same tumor** ^2^			
(FDR or SDR), number (%)	20 (44.4)	10 (31.2)	30 (38.9)
Number of affected relatives; median (IQR)	2 (1.2–3)	2 (1–2.7)	----
Family history of the same tumor in FDR, number (%)	18 (40)	8 (25)	26 (33.7)
Younger age at diagnosis; median (IQR)	55 (44.5–67.5)	62.5 (52.5–69.7)	-----
**Family history of other neoplasms** (FDR or SDR)	29 (64.4)	24 (75)	53 (68.8%)
Number of affected relatives; median (IQR)	1.5 (1–3)	2 (1–3)	2 (1–3)
Total registered tumors	65	56	121
Most frequent tumors, number (%)			
Breast	14 (21.5)	4 (7.1)	18 (14.8)
Colon	7 (10.7)	5 (8.9)	13 (10.7)
Lung	2 (3.1)	3 (5.3)	5 (4.1)
Prostate	3 (4.6)	3 (5.3)	6 (4.9)
Endometrium	1 (1.5)	3 (5.3)	4 (3.3)
Pancreas	5 (7.7)	-	5 (4.1)
Leukemia	1 (1.5)	3 (5.3)	4 (3.3)
**IHC-MMR tumor**, number (%)	29 (64.4)	9 (28.1)	38 (49.3)
**Altered IHC-MMR**, number (%)	1 (3.4)	0	1 (2.6)
**Germline genetic study**, number (%)	16 (35.6)	6 (18.8)	22 (28.6)
Gene-guided study (Sanger)	8 (50)	1 (16.7)	9 (40.1)
Multi-gen panel	8 (50)	5 (83.3)	13 (59.1)
**Germline genetic mutations identified**, number (%)	7 (43.7)	3 (18.8)	10 (30.3)
**Familial cancer criteria**, number (%)			19 (24.7)
Criteria of FGC	17 (37.8)	-	
≥3 FDR or SDR with GC	5 (29.4)	-	
≥2 FDR or SDR with GC, 1 < 50	7 (70.6)	-	
Criteria of FPC	-	7 (21.8)	
≥3 FDR/SDR/TDR with PC	-	2 (28.5)	
≥2 FDR with PC	-	5 (71.5)	
**Final classification after genetic counseling**, number (%)	7 (26.7)	3 (9.4)	10 (13)
(a) Hereditary-cancer associated syndrome			
(b) Familial form of cancer	12 (15.5)	7 (21.9)	19 (24.6)
(c) Sporadic cancer	26 (57.8)	22 (68.7)	48 (62.4)

IQR, interquartile range; PH, personal history; GC, gastric cancer; PC, pancreatic cancer; FDR, first-degree relative; SDR, second-degree relative; IHC-MMR, Immunohistochemical analysis of DNA repair proteins, FGC, familial gastric cancer; FPC, familial pancreatic cancer. ^1^ Age of the youngest GC/PC case in the family. ^2^ Family history of GC in case of PH of GC; and family history of PC in case of PH of PC.

**Table 2:** Characteristics of patients with hereditary cancer (germline genetic mutation identified).

*P*.	G.	Age ^1^	Gender	Referral Criteria	Personal History of Other Cancer	Family History of Cancer *	IHC-MMR	GeneticsTechnique	Gene Affected (Pathogenic Variant)	Hereditary Syndrome
1	GC	75	Female	GC+ other	Endometrium	No	MSH2/MSH6	Single-gene testing	MSH2 (c.602dupT p.Leu201Phe*31)	Lynch S
2	GC	55	Female	Family history	Breast	No	Undone	Multigen panel	*ATM (c.4507C>T (p.Gln1503Ter))*	ATM
3	GC	35	Female	Family history	Endometrium	Yes (pancreas and breast)	Undone	Multigen panel	*ATM (c.2921+1G>A. 19 intron)*	ATM
4	GC	41	Male	GC ≤ 60 years	No	No	Undone	Multigen panel	CDH1 (c.220C>T (p.Arg74 *))	HDGC
5	GC	38	Male	GC ≤ 60 years	No	No	Undone	Multigen panel	*CDH1 c.2164+5G>C)*	HDGC
6	GC	49	Female	GC ≤ 60 years	Breast	Yes (breast)	MMR+	Single-gene testing	*BRCA2 (c.3166 C>T))*	HBOC
7	GC	34	Male	GC ≤ 60 years	No	Yes (breast and colon)	MMR+	Multigen panel	*TP53 (c.365_366delTG (p.Val122AspfsTer26))*	Li-Fraumeni S
8	PC	45	Female	Family history	No	Yes (Breast, stomach)	Undone	Directed	BRCA2 c.3264dupT (p. (Gln1089Serfs*10),	HBOC
9	PC	33	Male	PC ≤ 60 years	No	Yes (breast)	Undone	Multigen panel	ATM (c.6711_6715delGGAAA (p.Lys2237Asnfs*10)	ATM
10	PC	33	Male	PC ≤ 60 years	No	Yes (breast)	Undone	Multigene panel	PALB2 (c.3483delT; p. (Phe1161Leufs*2))	HBOC

* Only hereditary syndrome-associated tumors are mentioned. *P*., patient; G., group; IHC-MMR, Immunohistochemical analysis of DNA repair proteins; GC, gastric cancer; PC, pancreatic cancer; MMR+, normal protein expression of DNA repair proteins; HC, hereditary cancer; HBOC, hereditary breast-ovarian cancer syndrome; S., Syndrome. ^1^ If no personal history of GC or PC, the age corresponds to the youngest relative.

**Table 3:** Analysis of risk factors for hereditary and familial gastric and pancreatic cancer. Univariate and multivariate analysis of factors associated with the detection of: a high-risk form (a); or hereditary syndrome (b).

**(a) Factors Associated with High Risk Condition of Cancer (Hereditary or Familial Cancer)**				
**Characteristic**	**Sporadic Cancer (48)**	**High Risk Form (29)**	***p*-Value (Univariate)**	**OR (95% CI); *p*-Value** **(Multivariate)**
Age, years; median (IQR)	53 (41.5–67)	49 (41.2–55.5)	0.084	0.98 (0.92–1.03); *p* = 0.46
Gender: women; number (%)	29 (60.4)	17 (58.6)	0.876	-
PH of other cancer	4 (8.3)	5 (17.2)	0.252	-
Referral criteria:				
Age ≤ 60 years old	42 (87.5)	9 (31)	0.000	0.16 (0.008–3.37); *p* = 0.24
Multiplicity	2 (4.1)	1 (3.4)	0.875	-
Family history	4 (8.3)	19 (65.5)	0.000	21 (5.8-79); *p* = 0.000
**(b) Factors Associated with Hereditary Cancer (Only Cases with Pathogenic Mutation Identified)**				
**Characteristic**	**Non-Hereditary Cancer (67)**	**Hereditary Syndrome (10)**	**P-Value (univariate)**	**OR (95% CI); *p*-Value (multivariate)**
Age, years; median (IQR)	50 (43–59)	39.5 (33.7–50.5)	0.041	
Younger case age < 40	10 (15)	5 (50)	0.009	11.3 (1.9–67); 0.007
Gender: women; number (%)	41 (61.2)	5 (50)	0.501	
PH of other cancer	5 (7.4)	4 (40)	0.003	17.4 (2.5–119.9); *p* = 0.004
Referral criteria:				
Age ≤ 60 years old	45 (67.2)	6 (60)	0.655	
Multiplicity	2 (2.9)	1 (10)	0.285	
Family history	20 (29.8)	3 (30)	0.992	

IQR, interquartile range; OR, odds ratio; CI, confidence interval; PH, personal history.
